# Dual‐Emulsifier Coated Photocatalyst for H_2_O_2_ Synthesis in Emulsion via Water Oxidation

**DOI:** 10.1002/advs.202517645

**Published:** 2025-10-28

**Authors:** Xueyang Leng, Wujun Zhang, Yangyang Lu, Lingling Xu, Yanbin Shen, Flemming Besenbacher, Emma Richards, Hong Gao, Ren Su

**Affiliations:** ^1^ Key Laboratory of Photonic and Electronic Bandgap Materials School of Physics and Electronic Engineering Harbin Normal University Harbin 150025 China; ^2^ Soochow Institute for Energy and Materials Innovations (SIEMIS) Soochow University Suzhou 215006 China; ^3^ Suzhou Institute of Nano‐Tech and Nano‐Bionics (SINANO) Chinese Academy of Sciences Suzhou 215123 China; ^4^ The Interdisciplinary Nanoscience Center (iNANO) Aarhus University Aarhus DK‐8000 Denmark; ^5^ School of Chemistry Cardiff University Park Place Cardiff CF10 3AT UK

**Keywords:** emulsion, H_2_O_2_ decomposition, H_2_O_2_ production, photocatalysis, water oxidation

## Abstract

Photocatalysis provides a sustainable approach for on‐site production of H_2_O_2_, yet single‐phase systems generally display unsatisfactory efficiency and low concentration of H_2_O_2_ due to rapid reverse reactions and dissociation of H_2_O_2_. Multiphase systems are developed to yield aqueous H_2_O_2_ via oxygen reduction by employing hydrophobic photocatalysts, however, the low solubility of oxygen and the possible dissociation of generated H_2_O_2_ result in a limited improvement in catalytic performances. Herein, a lauric acid (LA)‒*n*‐dodecyltrimethoxysilane (DTMS) dual‐emulsifier coated Pd/TiO_2_ (LD‐Pd/TiO_2_) is constructed for the synthesis of H_2_O_2_ in a water‐nonane system via water oxidation with quintozene as an insoluble hydrogen acceptor in water. While DTMS in the composite coating quenches the decomposition of H_2_O_2_, LA facilitates the enrichment of quintozene near the Pd/TiO_2_ for a rapid consumption of hydrogen atoms. The LD‐Pd/TiO_2_ leads to a remarkable H_2_O_2_ concentration of 133 mM with decent stability and a high quantum efficiency (6.5% at 365 nm). Additionally, the system can be demulsified gently after reaction, obtaining aqueous H_2_O_2_ solution and oil phase with hydrogenated products for simple separation and collection.

## Graphical abstract

A dual‐emulsifier coated photocatalyst facilitates efficient synthesis of H_2_O_2_ in water and the hydrogenation in oil via water dissociation.

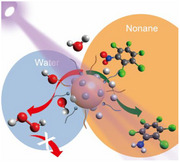

## 1 Introduction

Hydrogen peroxide (H_2_O_2_) is an essential chemical for disinfection, chemical synthesis, wastewater treatment, and bleaching,^[^ ^1^ ^]^ and is estimated to reach a global market of 5.7 million tons by 2028.^[^ ^2^ ^]^ Currently, more than 95% of the commercially available H_2_O_2_ is synthesized through the anthraquinone process that relies on the hydrogenation of anthraquinone and oxidation of anthrahydroquinone, resulting in significant energy consumption and waste emissions.^[^ ^3^ ^]^ Additionally, the storage, transportation, and handling of concentrated H_2_O_2_ pose significant safety issues. Since a dilute H_2_O_2_ solution is sufficient for most applications (i.e., ≈30 mm for water treatment and antibacterial applications),^[^ ^4^, ^5^ ^]^ photocatalytic H_2_O_2_ production from oxygen reduction and/or water oxidation for on‐site and on‐demand applications is considered as a sustainable and applicable solution.^[^ ^6^, ^7^, ^8^, ^9^ ^]^ A relatively high concentration of H_2_O_2_ can be produced via oxygen reduction with the presence of an alcohol molecule as the sacrificial agent.^[^ ^10^ ^]^ However, the separation and purification of photogenerated H_2_O_2_ remain a challenging issue, due to the high solubility of alcohol and their oxidized products (i.e., 42.9 and 6.95 g L^−1^ for benzyl alcohol and benzaldehyde).

**Scheme 1:**
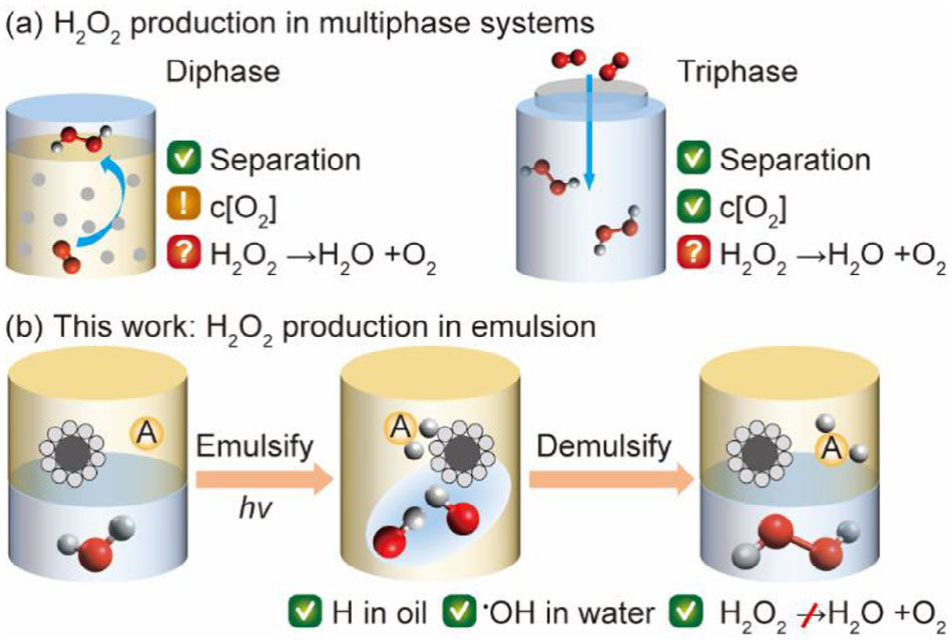
Strategies of photocatalytic H_2_O_2_ synthesis. a) Conventional multiphase systems from O_2_ reduction. b) This work: an emulsion system for H_2_O_2_ production from H_2_O oxidation with an insoluble hydrogen acceptor (A).

Conducting photocatalytic H_2_O_2_ in multiphase systems provides simultaneous separation of reactants and products, thus could solve the aforementioned issues in monophase systems (**Scheme** **1**a). This was first reported in the pioneering work of Yamashita, which employs a hydrophobic metal organic framework (MOF) photocatalyst for the reduction of molecular oxygen in an organic phase that consists of benzyl alcohol as a hole scavenger.^[^ ^11^ ^]^ While benzyl alcohol and the produced benzaldehyde predominantly remain in the organic phase,^[^ ^12^, ^13^, ^14^ ^]^ the photogenerated superoxide radicals diffuse to the aqueous phase to produce H_2_O_2_ by interacting with protons.^[^ ^15^ ^]^ Since then, a series of MOFs and covalent organic frameworks (COFs)‐based photocatalysts with engineered hydrophobic functional groups have been developed to further enhance the photocatalytic performance with the presence of selected alcohols.^[^ ^16^, ^17^, ^18^ ^]^ However, the efficiency of such processes is limited by the solubility of oxygen in the organic phase. In addition, the reactive oxygen species could also be consumed by reacting with the organic scavengers prior to escaping from the organic phase,^[^ ^19^ ^]^ resulting in a limited yield of H_2_O_2_. Yu et al. have further established a triphasic system to overcome this issue by employing a COF‐based photocatalyst for H_2_O_2_ synthesis in pure water.^[^ ^20^ ^]^ A high production rate is achieved, yet the final concentration of H_2_O_2_ remains low (≈1.5 mM) for applications. Nevertheless, complete switching‐off the dissociation of H_2_O_2_ seems a challenging task in most cases,^[^ ^21^ ^]^ thus restricting the synthesis of H_2_O_2_ to a limited concentration.

Upgrading the multi‐phase system to an emulsion system via water oxidation could be a solution to overcome the challenges in mass transfer of reactive species and suppression of H_2_O_2_ dissociation. A few works show that heterogeneous catalysis in Pickering emulsions display enhanced performances in oxidative hydration, hydrogenation, and epoxidation,^[^ ^22^, ^23^, ^24^, ^25^ ^]^ owing to a close contact between reactants and catalysts at the water‐oil interface.^[^ ^25^, ^26^ ^]^ Yu and coauthors summarized recent developments in the construction of Pickering emulsions for photocatalytic applications, which predominantly focus on chemical conversions into stable products (i.e., pollutant decomposition, H_2_ evolution, and CO_2_ reduction).^[^ ^27^ ^]^ However, additional criteria need to be considered for the synthesis of meta‐stable H_2_O_2_ in emulsions from photocatalytic water oxidation (Scheme 1b). The process involves the dissociation of water into H atoms and hydroxyl radicals (^•^OH) on the surface of the photocatalyst, and the formation of H_2_O_2_ via the combination of two ^•^OH free radicals.^[^ ^28^, ^29^, ^30^ ^]^ Meanwhile, the generated surface adsorbed H atoms are preferably consumed by a hydrogen acceptor (**A**) to push the reaction equilibrium forward. Therefore, it is crucial to formulate an emulsion to **i**) insulate the generated H_2_O_2_ from the photocatalyst to avoid dissociation; **ii**) accumulate **A** molecules in the oil phase adjacent to the photocatalyst for subsequent hydrogenation; and **iii**) remove the generated H atoms from the photocatalyst and the aqueous phase transferring them into the oil phase to switch‐off unwanted reverse reactions with ^•^OH radicals. Additionally, it should also be simple to demulsify to obtain an aqueous H_2_O_2_ solution and the reduction product in the oil phase, thus benefiting the collection of pure H_2_O_2_ solution by employing a water‐insoluble hydrogen acceptor.

Here, we have rationally designed a dual‐emulsifier coated Pd/TiO_2_ photocatalyst to realize efficient synthesis of H_2_O_2_ from water oxidation with quintozene as the hydrogen acceptor (**A**) in a water‐nonane mixture. By evaluating some commonly used emulsifiers in suppressing catalytic H_2_O_2_ dissociation and promoting photocatalytic hydrogenation of A, we show that lauric acid (LA)‒n‐dodecyltrimethoxysilane (DTMS) coated Pd/TiO_2_ (LD‐Pd/TiO_2_) achieves a remarkable H_2_O_2_ concentration of 133 mM, a high quantum efficiency, and an excellent stability. The emulsion system can be demulsified by a mild centrifugation, yielding aqueous H_2_O_2_ solution and oil phase with hydrogenated products for simple separation and collection. Kinetic analysis and the evolution of trapped radical species are performed to probe the promotional mechanisms of photocatalyzed H_2_O_2_ production in emulsion.

## 2 Results and Discussion

### 2.1 The Functions of Emulsifiers

**Figure 1:**
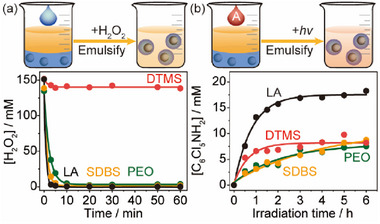
Searching for functional emulsifiers. a) Inhibition in catalytic dissociation of H_2_O_2_ and b) acceleration in photocatalytic hydrogenation of quintozene with different emulsifiers. Reaction conditions: 10 mg emulsifier in 0.5 mL water and 1.5 mL nonane with 15 mg Pd/TiO_2_ and 40 mM KOH under 1 bar N_2_ at RT. 30 wt.% H_2_O_2_ or 100 mm quintozene is added prior to reaction. A 365 nm LED (38 mW cm^‒2^) is used for photocatalysis.

We have first examined four representative emulsifiers in preventing the dissociation of H_2_O_2_ and accelerating the photocatalytic hydrogenation of quintozene, by dosing concentrated H_2_O_2_ aqueous solution (30 wt.%) and quintozene separately into water‐nonane systems that contains Pd/TiO_2_ catalyst (**Figure** **1**a,b; Note S1, Supporting Information). A direct comparison of the emulsifiers in inhibiting H_2_O_2_ dissociation is shown in Movie S1 (Supporting Information). Remarkably, the addition of DTMS into the water‐nonane‐Pd/TiO_2_ system shuts down the dissociation of added H_2_O_2_ completely (Figure 1a). Surprisingly, the addition of LA, sodium dodecyl benzene sulfonate (SDBS), and polyethylene oxide (PEO) all fail to avoid the rapid and complete dissociation of H_2_O_2_. It is therefore proposed that DTMS insulates the Pd nanoparticles (NPs) from H_2_O_2_ molecules, which otherwise act as an efficient catalyst for the decomposition of H_2_O_2_ (Figures S1 and S2, Supporting Information).^[^ ^31^ ^]^ Interestingly, a rapid formation of pentachloroaniline with a relatively high yield is only observed when adding LA into the water‐nonane system that contains Pd/TiO_2_ powders (Figure 1b). In comparison, replacing LA with DTMS, PEO, and SDBS results in a slow formation rate of pentachloroaniline with a low yield (Figures S3 and S4, Supporting Information). Since the photogenerated hydrogen atoms are preferentially adsorbed on Pd sites for hydrogenation,^[^ ^32^ ^]^ it is considered that the addition of LA as the emulsifier facilitates the enrichment of quintozene close to the Pd NPs.

### 2.2 The Coated Photocatalyst

**Figure 2:**
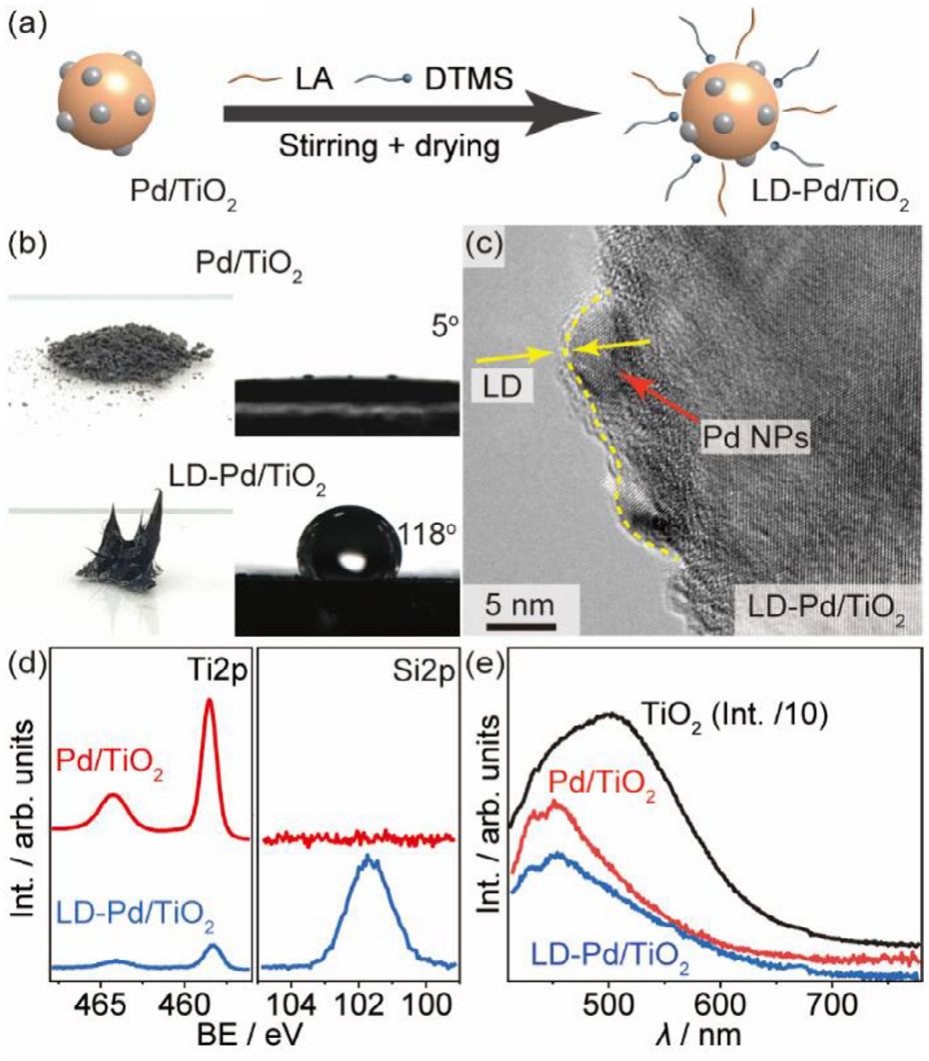
Synthesis and characterizations of the LD‐Pd/TiO_2_. a) Coating procedure of the uncoated Pd/TiO_2_. b–e) Optical imaging, contact angle, TEM imaging, XPS, and PL spectra of the LD‐Pd/TiO_2_ in comparison with Pd/TiO_2_.

It is considered that an optimum photocatalytic performance could be achieved by inheriting the merits of both LA and DTMS. Therefore, we have coated Pd/TiO_2_ with LA and DTMS (LD‐Pd/TiO_2_) through a simple mixing and drying process under ambient conditions (**Figure** **2**a; Note S2, Supporting Information). Fluorescence microscopic imaging reveals that the dual emulsifier coating inherits the merits of individual emulsifier (Figure S5, Supporting Information), which facilitates the formation of an optimum water‐oil‐solid triple phase but prevents the invasion of solid photocatalyst into water. The wrapping of Pd/TiO_2_ by LA and DTMS results in an agglomeration of the original Pd/TiO_2_ gray powders and a drastic change of contact angle (Figure 2b), revealing an evolution from hydrophilic to hydrophobic surface. The coating also leads to an amorphous thin layer on both the Pd NPs and the crystalline TiO_2_ according to transmission electron microscopy imaging (TEM, Figure 2c; Figure S6, Supporting Information). The Pd NPs displays an average particle size of ≈3.3 nm, and the emulsifier coating barely alternate the particle size of the Pd NPs (Figure S6, Supporting Information). The homogeneous covering of the DTMS and LA is also confirmed by energy dispersive spectroscopy mapping (EDS, Figure S7, Supporting Information). X‐ray photoelectron spectroscopy analysis further validates the surface modification after the coating procedure (XPS, Note S3, Supporting Information). The intensity of Ti2p peaks decreases drastically but does not vanish completely, accompanied with the presence of an intense Si2p peak (Figure 2d), suggesting the formation of a thin layer (<5 nm) assembled by emulsifiers.^[^ ^33^ ^]^ The disappearance of the Pd3d peaks after coating is due to the low loading of Pd NPs (≈1.3 wt.%, Figure S8 and Table S1, Supporting Information), rendering only weak signals in the original uncoated materials. The narrow thickness of the emulsifier layer ensures only a minimum negative impact for the interfacial transfer of photogenerated charge carriers, according to a previous investigation on the mean‐free path of excited electrons.^[^ ^34^ ^]^ This has been evidenced by the photoluminescence spectra (PL, Figure 2e), where the LD‐Pd/TiO_2_ displays a similar peak intensity to the uncoated Pd/TiO_2,_ indicating no appreciable changes in charge recombination. Both Pd‐decorated materials present a significantly reduced PL than the pristine TiO_2_ as anticipated.^[^ ^35^ ^]^ A blue shift of the emission peak observed for both Pd‐decorated TiO_2_ is associated to the reduced population of excited electrons at trap states below the conduction band minimum (CBM) of TiO_2_ due to the introduction of Pd NPs, which hold the excited electrons at the apparent Fermi level that is slightly lower than the CBM of TiO_2_. The lifetimes of photogenerated charge carries for TiO_2_, Pd/TiO_2_, and LD‐Pd/TiO_2_ are very close according to time‐resolved PL (Figure S9, Supporting Information), suggesting that the presence of Pd or emulsifier coating barely alternates the kinetics of charge separation. Additionally, the coating barely influences the physical properties of the Pd/TiO_2_ according to X‐ray diffraction (XRD), infrared spectroscopy, and diffuse reflectance spectroscopy (DRS), respectively (Figure S10, Supporting Information). A negative shift (−0.4 eV) of both CBM and valence band maximum (VBM) is observed for emulsifier coated photocatalyst (Figure S10, Supporting Information). This is possibly due to an uneven distribution of electrons at the interface caused by the long‐chain alkyl groups of the emulsifier, which generates a dipole moment that affects the distribution of the electric field on the Pd/TiO_2_ surface.^[^ ^36^ ^]^ The negatively shifted VBM and CBM of the LD‐Pd/TiO_2_ matches well with the required redox potentials for the oxidation of OH^‒^/^•^OH and hydrogenation of quintozene, thus are thermodynamically favorable to complete the reaction.

### 2.3 Catalytic Performance

**Figure 3:**
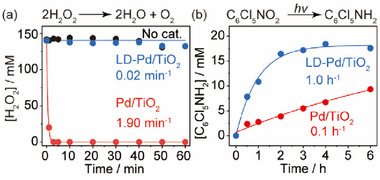
Performance and kinetics. a) and b) Kinetics of H_2_O_2_ dissociation and photocatalytic hydrogenation of quintozene in a water‐nonane system using LD‐Pd/TiO_2_ and Pd/TiO_2_.

We have first validated the capability of LD‐Pd/TiO_2_ in inhibiting H_2_O_2_ decomposition and accelerating hydrogenation of quintozene (**Figure** **3**a,b). While a rapid depletion of H_2_O_2_ is observed for Pd/TiO_2_ in basic media under ambient conditions, >95% of the original H_2_O_2_ is well preserved when LD‐Pd/TiO_2_ is employed as the catalyst. The negligible reduction of H_2_O_2_ can be attributed to the self‐decomposition of H_2_O_2_ upon comparison of similar behavior observed in the complete absence of a catalyst. A real‐time video directly compares the distinct decomposition rates of concentrated H_2_O_2_ using LD‐Pd/TiO_2_ and Pd/TiO_2_ in alkaline conditions (Movie S2, Supporting Information). Meanwhile, the LD‐Pd/TiO_2_ also displays a much better performance in photocatalytic hydrogenation of quintozene than the Pd/TiO_2_ (Figure 3b), which is similar to that of Pd/TiO_2_ with dispersed LA in emulsion (Figure 1b). It suggests that the dual LA‐DTMS coating on Pd/TiO_2_ succeeds in combining and utilizing the functions of both LA and DTMS dispersed in emulsions.

**Figure 4:**
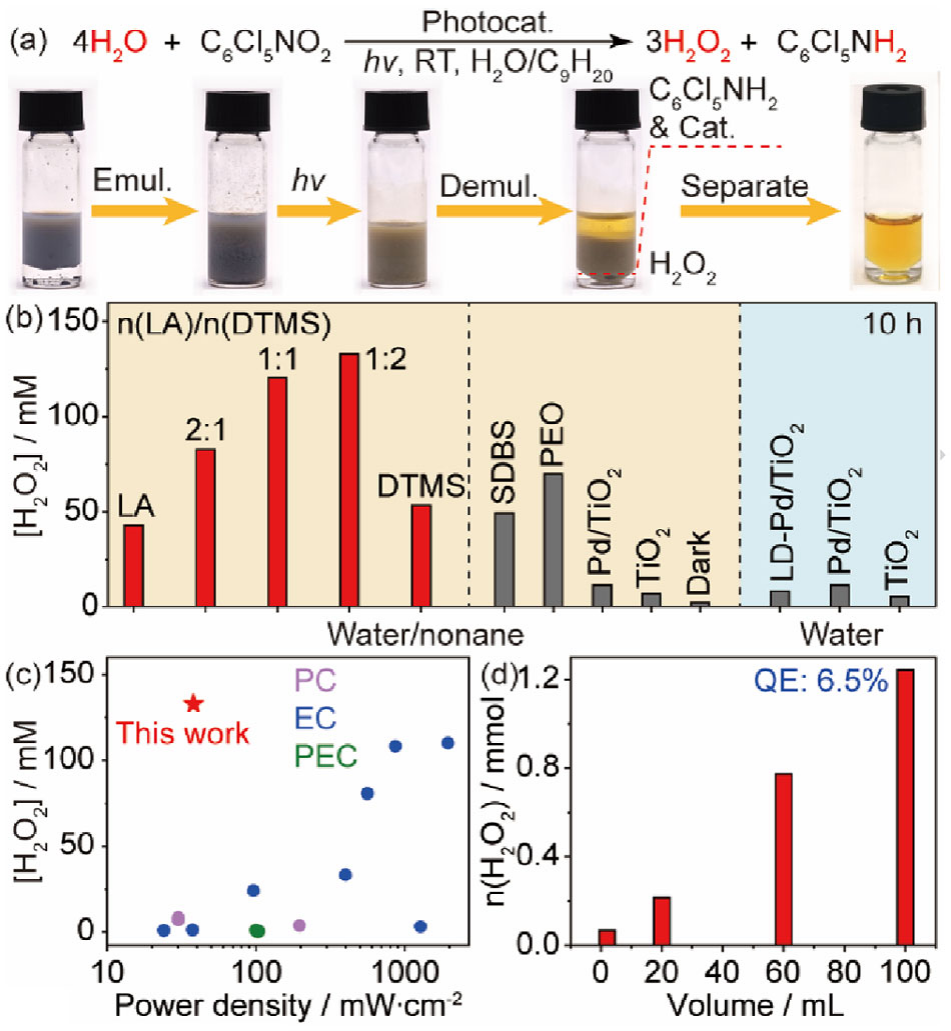
Performance and kinetics. a) and b) Images and performance of photocatalytic H_2_O_2_ production from water dissociation using LD‐Pd/TiO_2_ in emulsion. Standard reaction conditions: 15 mg photocatalyst in 0.5 mL water and 1.5 mL nonane with 100 mm quintozene, 1.7 vol.% 1,4‐dioxane, and 40 mM KOH under 365 nm irradiation (38 mW cm^‒2^) and 1 bar N_2_ at RT for 10 h. c) Comparison with reported H_2_O_2_ production systems via water oxidation. PC: photocatalysis; EC: electrocatalysis; PEC: photoelectrolysis. d) Scalability of H_2_O_2_ evolution in emulsion.

Photocatalytic synthesis of H_2_O_2_ from water oxidation was performed in a water‐nonane mixture using LD‐Pd/TiO_2_ with a 365 nm LED (38 mW cm^‒2^) under basic (40 mm KOH) and deaerated conditions at room temperature (RT), as illustrated in **Figure** **4**a. Quintozene (100 mM) and LD‐Pd/TiO_2_ (15 mg) are well dispersed in nonane under static conditions, due to their insolubility in water and hydrophobicity. Meanwhile, 1,4‐dioxane (1.7 vol.%) as the hydroxyl mediator (**M**) is dissolved in both nonane and water phases.^[^ ^37^ ^]^ Quintozene is used as the hydrogen acceptor owing to its low solubility in water (0.44 mg L^−1^), which is essential to obtain an aqueous H_2_O_2_ solution with a relatively high purity. Additionally, the reduction product (pentachloroaniline) is also insoluble in water (1.332 mg L^−1^), facilitating the collection and regeneration of chemicals. The two phases turn into an emulsion under mild stirring, enabling photocatalytic reactions in a homogeneous system upon irradiation. The emulsion turns back to a two‐phase system ≈10 min after switching off the stirring, or via a gentle centrifugation. The hydrophobic LD‐Pd/TiO_2_, the insoluble quintozene, and reduced pentachloroaniline enable an easy separation of the generated aqueous H_2_O_2_ solution at the bottom of the reaction vial. Upon a gentle rotary evaporation, high purity nonane and pentachloroaniline (Figure S11, Supporting Information) can be obtained, featuring a high sustainability of our process. Additionally, the pentachloroaniline in nonane can be oxidized back to quintozene under mild conditions for recycling.^[^ ^38^ ^]^

The composition and identity of the emulsifier coating on Pd/TiO_2_ is crucial in achieving a high photocatalytic performance for H_2_O_2_ production from water oxidation in emulsion (Figure 4b). Either single LA or DTMS coated Pd/TiO_2_ displays a much lower yield of H_2_O_2_ than the LA‐DTMS composite coated Pd/TiO_2_, and an optimum performance is observed at a LA/DTMS molar ratio of 1:2. Remarkably, the concentration of H_2_O_2_ in aqueous solution reaches ≈133 mM within an irradiation time of 10 h under optimized reaction conditions (Figure 4b; Figure S12, Table S2 and Note S4, Supporting Information). This is attributed to an acceleration of photocatalytic quintozene hydrogenation and an optimum suppression of catalytic H_2_O_2_ dissociation in emulsion. The quantity of LA and DTMS influences the thickness of the emulsifier coating on Pd/TiO_2_, thus manipulating the kinetics of quintozene hydrogenation and H_2_O_2_ dissociation and eventually achieving an optimum performance (Figure S13, Supporting Information). An optimum loading of LA and DTMS is also essential in balancing the inhibition of H_2_O_2_ dissociation and water adsorption (Figure S14, Supporting Information). No reaction was observed under dark conditions, confirming that the generated H_2_O_2_ was solely derived from photocatalytic water oxidation. Employing SDBS and PEO as coating only enhances the H_2_O_2_ production to some extent. As expected, the combination of DTMS‐PEO and LA‐SDBS all show a reduced H_2_O_2_ production, due to a limited efficiency in inhibiting H_2_O_2_ decomposition and hydrogenation (Figure S15, Supporting Information). In comparison, the concentration of H_2_O_2_ only reaches ≈10 mM when employing Pd/TiO_2_ and pristine TiO_2_ as photocatalyst, revealing the essential role of the emulsifier coating in promoting H_2_O_2_ evolution. As expected, the absence of quintozene leads to a negligible performance, due to the unwanted reversible reaction of hydrogen atoms with hydroxyl radicals. In comparison, poor performances are observed for all three photocatalysts in water without nonane, due to the poor solubility of quintozene in water that restricts the mass transfer of photogenerated hydrogen atoms. The LD‐Pd/TiO_2_ outperforms a series of coated metal/TiO_2_ in H_2_O_2_ evolution (Figure S16, Supporting Information), owing to an optimum adsorption energy of photogenerated hydrogen atoms for the hydrogenation of quintozene.^[^ ^32^ ^]^ The optimum LD‐Pd/TiO_2_ photocatalyst displays a superior performance in H_2_O_2_ production at a low power density via water oxidation, in comparison with reported works by photo‐, electro‐, and photoelectro‐ catalysis (Figure 4c; Table S3, Supporting Information).^[^ ^37^, ^39^, ^40^, ^41^, ^42^, ^43^, ^44^, ^45^, ^46^, ^47^, ^48^, ^49^, ^50^, ^51^, ^52^ ^]^ Importantly, a linear increase of photogenerated H_2_O_2_ in quantity is observed as the reaction volume is scaled up from 2 to 100 mL at a fixed irradiation time of 10 h (Figure 4d), leading to an optimum quantum efficiency (QE) of 6.5% and a yield of 1.2 mmol for H_2_O_2_ at 100 mL (Note S5, Supporting Information).

### 2.4 Reusability and Stability

**Figure 5:**
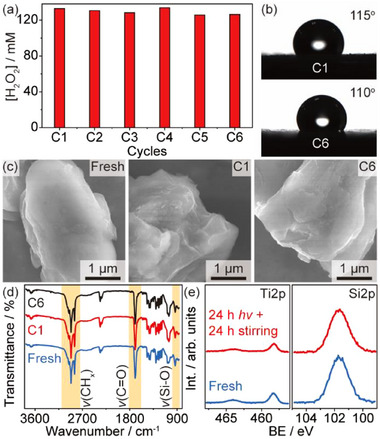
Stability evaluation. a) Photocatalytic generation of H_2_O_2_ using LD‐Pd/TiO_2_ in emulsion for six consecutive cycles. Reaction conditions: 15 mg photocatalyst in 2 mL nonane/water (3:1 v v^−1^), 1.7 vol.% dioxane, and 40 mm KOH with 100 mM C_6_Cl_5_NO_2_ under 365 nm irradiation (38 mW cm^−2^) and 1 bar N_2_ at RT for 10 h. b–d) Contact angle, SEM imaging, and FTIR of the fresh and spent LD‐Pd/TiO_2_. e) XPS of the fresh and spent LD‐Pd/TiO_2_ after 24 h of continuous irradiation and 24 h of immersion in emulsion under continuous stirring.

We have evaluated the stability of the LD‐Pd/TiO_2_ by directly collecting and recycling the spent photocatalyst without any intermediate processing. The high performance of the LD‐Pd/TiO_2_ remains unchanged over six consecutive cycles, revealing a superior stability of the emulsifier coating (**Figure** **5**a). This is confirmed according to the contact angle of the spent LD‐Pd/TiO_2_, which only slightly reduced to 115° after the 1st use (C1) and stabilized at ≈110° after the 6th run (C6, Figure 5b). The fresh and cycled LD‐Pd/TiO_2_ is well wrapped by the emulsifier coating throughout the test, as validated by SEM imaging (Figure 5c), which is essential to maintain a high catalytic performance. The homogeneous distribution of Pd NPs and the LD coating layer also remain unchanged after photocatalytic H_2_O_2_ evolution according to TEM and EDS mapping analysis (Figure S17, Supporting Information), confirming the excellent stability of the photocatalyst. Meanwhile, no leaching of LA and DTMS is observed according to gas chromatography (GC) analysis of the liquid phase (Figure S18, Supporting Information). Additionally, infrared spectroscopy shows that the C─H, C═O, and Si─O vibrational peaks of the cycled LD‐Pd/TiO_2_ remain as is (Figure 5d), revealing that both LA and DTMS molecules anchored on Pd/TiO_2_ are well‐preserved during the formation of H_2_O_2_. This is further validated by solid state nuclear magnetic resonance (ssNMR) analysis of the fresh and spent emulsifier coated Pd/TiO_2_ (Figure S19, Supporting Information). Remarkably, the peak intensity of Ti2p and Si2p and the Si2p/Ti2p ratio of the spent LD‐Pd/TiO_2_ after a continuous irradiation (24 h) and immersion (24 h) in emulsion are comparable with the fresh LD‐Pd/TiO_2_ (Figure 5e), revealing the feasibility for long‐term operations. A slightly reduced Pd loading of the spent photocatalyst is associated to aggressive sonication prior to ICP‐AES analysis rather than leaching during photocatalysis, as the loading of Pd remains constant using an improved preparation method for characterization (Table S1, Supporting Information).

### 2.5 Promotional Mechanisms

**Figure 6:**
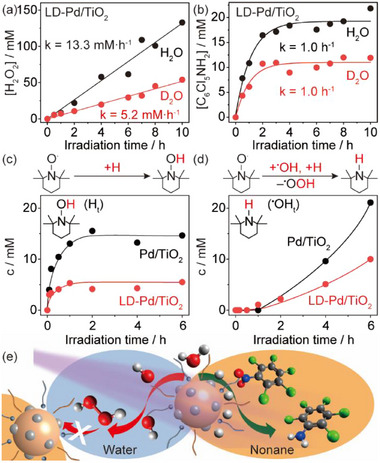
Promotional mechanisms. a) and b) Isotopic kinetics of photocatalytic H_2_O_2_ and pentachloroaniline evolution in emulsion from H_2_O and D_2_O. c) and d) Evolution of trapped ^•^OH (^•^OH_t_) and H (H_t_) in aqueous phase by TEMPO. e) Enhanced photocatalytic H_2_O_2_ production in emulsion by LD‐Pd/TiO_2_.

The photocatalytic production of H_2_O_2_ from water oxidation only proceeds under a basic media (Figure S20, Supporting Information), indicating an indirect water oxidation pathway for the generation of ^•^OH radicals from OH^‒^ anions (1.99 V vs reversible hydrogen electrode, RHE).^[^ ^49^ ^]^ The alkaline environment (pH >13) also inhibits the evolution of molecular hydrogen to facilitate the hydrogenation of quintozene. A reduced H_2_O_2_ evolution is noticed under aerobic conditions, due to the competition of quintozene reduction with oxygen reduction (Figure S21, Supporting Information), ruling out H_2_O_2_ production via oxygen reduction. Time profiling shows that the photocatalytic evolution of H_2_O_2_ in emulsion follows a 0th‐order kinetics with a rate constant of 13.3 mM h^−1^, (**Figure** **6**a). The 0th‐order kinetics imply that the rate limiting step is the water oxidation restrained by the density of active sites, rather than the transfer and recombination of the hydroxyl radicals to yield H_2_O_2_. This is further verified by replacing H_2_O with D_2_O, where a significant primary isotopic effect of 2.56 is observed. Meanwhile, a *pseudo* 1st‐order kinetics with a negligible isotopic effect is observed for the formation of pentachloroaniline, revealing that the hydrogenation half reaction is mainly dependent on the diffusion of quintozene into the Pd active sites to accept the H atoms (Figure 6b). A lower concentration of deuterated pentachloroaniline is associated to a reduced quantity of atomic deuterium from the dissociation of D_2_O. In addition, labelled 1,4‐dioxane‐2‐ol with a molar mass of 105 (C_4_O_2_H_7_OD) and 106 (C_4_O_2_H_7_^18^OH) are observed when D_2_O and H_2_^18^O are used for photocatalysis under an acidic environment (Figure S22, Supporting Information). Since the stable 1,4‐dioxane‐2‐ol is derived from the hydroxylated dioxane mediator,^[^ ^37^ ^]^ it indicates that the generated ^•^OD and ^•18^OH radicals are solely from water oxidation. The use of dioxane as a hydroxyl mediator regulates its reaction kinetics, resulting in a slow formation rate of H_2_O_2_ than the hydrogenation rate of quintozene. This can be accelerated by using a higher concentration of dioxane (Figure S23, Supporting Information), where the evolution rates of H_2_O_2_ from water oxidation and pentachloroaniline from quintozene reduction tend to be synchronized.

The LA‐DTMS composite coating also plays a crucial role in modulating the accumulation of photogenerated ^•^OH radicals and H atoms, according to the evolution of captured radicals by 2,2,6,6‐tetramethylpiperidine 1‐oxyl (TEMPO, Figure 6c,d; Figure S24 and Note S6, Supporting Information).^[^ ^53^ ^]^ Remarkably, a significantly low concentration of trapped H atoms (H_t_) in the aqueous phase is observed for LD‐Pd/TiO_2_, whereas the concentration of H_t_ in the aqueous phase remains high when employing pristine Pd/TiO_2_ (Figure 6c). Additionally, the LD‐Pd/TiO_2_ shows a slower built‐up and lower maximum concentration of trapped ^•^OH radicals (^•^OH_t_) in the aqueous phase than the pristine Pd/TiO_2_, implying an efficient conversion of ^•^OH radicals into H_2_O_2_ and a suppression of any subsequent H_2_O_2_ dissociation. The promotional mechanisms of LD‐Pd/TiO_2_ in photocatalytic H_2_O_2_ production via water oxidation is therefore demonstrated in Figure 6e. Since both LA and DTMS display water‐in‐oil characteristics, an emulsion with LD‐Pd/TiO_2_ surrounded by water as the inner layer and nonane as the outer layer is formed under stirring. Upon irradiation, the surface adsorbed water molecule is dissociated into ^•^OH radicals and H atoms. The photogenerated ^•^OH radicals recombine into H_2_O_2_ in the aqueous phase, whereas the photogenerated H atoms rapidly diffuse into and are maintained in the oil phase. This not only benefits the spatial separation of ^•^OH radicals and H atoms, but also facilitates the consumption of H atoms by quintozene, which is soluble in nonane but insoluble in water. Additionally, the spatial separations of the photogenerated H_2_O_2_ from Pd NPs prevents the unwanted dissociation of H_2_O_2_.

The promotional mechanism is further probed by employing LA and DTMS coated Pd/TiO_2_ (LA‐Pd/TiO_2_ and LA‐Pd/TiO_2_). While LA forms a discrete, plate‐like layer inserted on the photocatalyst, DTMS produces a homogenous coating that completely wraps the Pd/TiO_2_ (Figures S8 and S25, Supporting Information). The outward LA plates and partially exposed Pd/TiO_2_ in LA‐Pd/TiO_2_ result in a more hydrophobic surface with enhanced interaction of non‐polar hydrogen acceptor (quintozene) according to FTIR and CA analysis (Figures S26 and S27, Supporting Information), thus facilitating the transfer of photogenerated hydrogen atoms. In contrast, the DTMS‐Pd/TiO_2_ exhibits a weak but distinguishable OH vibrational peak upon dosing water, indicating that a thin but homogeneous DTMS coating still allows the adsorption of water on the photocatalyst for further reactions. Notably, a physical isolation of Pd active sites and a significantly increased CA of aqueous H_2_O_2_ solution on DTMS‐Pd/TiO_2_ (130°) leads to a complete quenching of H_2_O_2_ dissociation. Therefore, we propose that DTMS tends to form a homogeneous coating on Pd/TiO_2_ with a reasonable permeability of water, allowing water dissociation for the generation of H atoms and ^•^OH radicals upon irradiation. The addition of LA in the coating facilitates the adsorption of quintozene on the photocatalyst, promoting the transfer of photogenerated hydrogen atoms from the Pd sites to quintozene. The ^•^OH radicals are recombined to produce H_2_O_2_ molecules, which are repelled by the DTMS layer to the aqueous phase.

## 3 Conclusion

In summary, we present the design of a dual‐emulsifier coated Pd/TiO_2_ (LD‐Pd/TiO_2_) for photocatalytic H_2_O_2_ evolution from water oxidation in a water‐nonane system with quintozene as the hydrogen acceptor. The combination of lauric acid (LA) and *n*‐dodecyltrimethoxysilane (DTMS) in the coating allows emulsification of the two‐phase system for efficient separation and transfer of photogenerated active intermediates under reaction conditions, while demulsifies after the reaction to yield aqueous H_2_O_2_ solution and oil phase with hydrogenated products for simple separation and collection. The LD‐Pd/TiO_2_ photocatalyst displays a remarkable performance in producing a high concentration of H_2_O_2_ up to 133 mM with a high quantum efficiency of 6.5% at a 100 mL scale under 365 nm irradiation, owing to an instant consumption of generated hydrogen atoms for hydrogenation and the suppression of H_2_O_2_ decomposition in the emulsion. The aqueous H_2_O_2_ solution with a trace quantity of dioxane could be employed for catalytic epoxidation, oxidation, and hydroxylation reactions. The LD‐Pd/TiO_2_ also shows excellent stability owing to the durable coating attached to the Pd/TiO_2_.

## Conflict of Interest

The authors declare no conflict of interest.

## Supporting information



Supporting Information

Supplemental Movie 1

Supplemental Movie 2

## Data Availability

The data that support the findings of this study are available in the supplementary material of this article.
