# Supplementary material for: Dual‐Emulsifier Coated Photocatalyst for H2O2 Synthesis in Emulsion via Water Oxidation
Source: Adv Sci (Weinh). 2025 Oct 28;13(3):e17645. doi: 10.1002/advs.202517645 (PMC12806344; doi:10.1002/advs.202517645)
Supplement: Supplementary file 1 — Supporting Information [file ADVS-13-e17645-s003.docx]

Supporting Information
©Wiley-VCH 2021
69451 Weinheim, Germany

Dual-emulsifier Coated Photocatalyst for H_2_O_2_ Synthesis in Emulsion via Water Oxidation

Xueyang Leng^#[a,b]^, Wujun Zhang^#[c]^, Yangyang Lu^[b]^, Lingling Xu^[a]^, Yanbin Shen^[c]^, Flemming Besenbacher^[d]^, Emma Richards*^[e]^, Hong Gao*^[a]^, and Ren Su*^[b]^

**Abstract:** Photocatalysis provides a sustainable approach for on-site production of H_2_O_2_, yet single-phase systems generally display unsatisfactory efficiency and low concentration of H_2_O_2_ due to rapid reverse reactions and dissociation of H_2_O_2_. Multiphase systems are developed to yield aqueous H_2_O_2_ *via* oxygen reduction by employing hydrophobic photocatalysts, however, the low solubility of oxygen and the possible dissociation of generated H_2_O_2_ result in a limited improvement in catalytic performances. Herein, we have constructed a lauric acid (LA)‒*n*-dodecyltrimethoxysilane (DTMS) dual-emulsifier coated Pd/TiO_2_ (LD-Pd/TiO_2_) for the synthesis of H_2_O_2_ in a water-nonane system *via* water oxidation with quintozene as an insoluble hydrogen acceptor in water. While DTMS in the composite coating quenches the decomposition of H_2_O_2_, LA facilitates the enrichment of quintozene near the Pd/TiO_2_ for a rapid consumption of hydrogen atoms. The LD-Pd/TiO_2_ leads to a remarkable H_2_O_2_ concentration of 133 mM with decent stability and a high quantum efficiency (6.5% at 365 nm). Additionally, the system can be demulsified gently after reaction, obtaining aqueous H_2_O_2_ solution and oil phase with hydrogenated products for simple separation and collection.

DOI: 10.1002/anie.2021XXXXX

Table of Contents

Experimental procedures 2

Supplementary movies 5

Supplementary tables 6

Supplementary figures 8

Supplementary references 12

Experimental Procedures

**Note S1. Exploration of functional emulsifier**

**Evaluation of emulsifiers in suppression of H_2_O_2_ decomposition:** A mixture of 75 μL KOH (4 M), 510 μL dioxane, 7.5 mL deionized (DI) water, and 22.5 mL nonane was added into a 50 mL beaker. Subsequently, 15 mg Pd/TiO_2_ catalyst and 10 mg emulsifier were added into the liquid under continuous stirring to form a stable emulsion. A 115 μL of concentrated H_2_O_2_ aqueous solution was then added into the emulsion at room temperature (RT), and a 2 mL of emulsion was extracted and centrifuged at given time intervals to obtain the aqueous phase. The concentration of remaining H_2_O_2_ in aqueous phase was quantitatively analyzed by a colorimetric titration method employing a Cu(II) based chromogenic agent.^[1]^

The chromogenic agent was prepared by mixing a 2,9-dimethyl-1,10-phenanthroline (DMP)-ethanol solution (1 g·L^−1^) with an aqueous CuSO_4_ solution (0.01 M) in a 1:1 volume ratio. For analysis, 50 μL of aliquots from the reaction were added into 3 mL of the freshly prepared chromogenic agent solution. The absorption spectra of the solution were measured by a UV-vis spectrophotometer (UV 1900i, Shimadzu) to determine the concentration of H_2_O_2_. The molar absorption coefficient (α) of the reduced chromogenic agent (Cu(I)-DMP complex) by H_2_O_2_ at 454 nm is determined to be 1 mM^-1^·cm^−1^ according to the calibration curve using a series of standard H_2_O_2_ solution with known concentrations (Fig. S1). Note that one part of H_2_O_2_ can reduce two parts of Cu(II)-DMP into Cu(I)-DMP.

The results are shown in Fig. S2. A real-time video directly compares the effect of different emulsifiers in inhibiting the decomposition of H_2_O_2_ (Movie S1).

**Evaluation of emulsifiers in promoting the hydrogenation of quintozene:** A mixture of 5 μL KOH (4 M), 34 μL dioxane, 0.5 mL DI water, and 1.5 mL nonane was added into a 4 mL glass reactor. Subsequently, 0.2 mmol quintozene (C_6_Cl_5_NO_2_), 15 mg Pd/TiO_2_ catalyst, and 10 mg emulsifier were added into the liquid under continuous stirring to form a stable emulsion. The emulsion was purged for 1.5 min by nitrogen gas to remove oxygen. A 365 nm annular LED was used as the light source (38 mW·cm^-2^). The emulsion was centrifuged at desired irradiation times, and the oil phase was extracted to analyze the remaining quintozene and produced pentachloroaniline by gas chromatography (GC, Agilent 8860) and gas chromatography-mass spectrometry (GC-MS, Agilent 8860 GC coupled with a 5977B mass selective detector). The calibration curve and results are shown in Figs. S3 and S4.

**Note S2. Synthetic protocols of photocatalysts**

All reagents and solvents were used without further purification unless otherwise stated.

**Pd/TiO_2_:** A modified photo-deposition method was utilized to prepare the Pd/TiO_2_.^[2]^ Firstly, 0.5 g of TiO_2_ and 16.6 mg of PdCl_2_ were added into 15 mL isopropanol and 10 mL DI water, aiming at a Pd loading of 2 wt%. The slurry was deaerated by N_2_ gas for three times under continuous magnetic stirring. A LED (365 nm, 38 mW·cm^−2^) lamp was then employed to irradiate the suspension at RT for 2 h under deaerated conditions. The Pd/TiO_2_ suspension was centrifuged and washed with DI water and ethanol for 3 times, and finally the powders were dried in an oven at 60 °C for 12 h.

**LD-Pd/TiO_2_:** 0.2 g lauric acid (LA, 1 mmol) and 0.6 g dodecyltrimethoxysilane (DTMS, 2 mmol) were dissolved in 40 mL H_2_O and stirred for 20 min at RT. Then 0.6 g Pd/TiO_2_ powders were added into the suspension under continuous stirring for 5 min. The resulting powders were centrifuged and dried in an oven at 60 °C for 12 h. Additionally, The LD-Pd/TiO_2_ photocatalysts with different molar ratios of LA/DTMS were synthesized by adjusting the added quantity of LA and DTMS.

**Note S3. Characterizations**

The hydrophobicity of the photocatalysts were determined with a JC2000D6 contact angle meter. Transmission electron microscopy (TEM, Themis Z, Thermo Fisher Scientific) was employed to analyze the morphology of Pd/TiO_2_ and LD-Pd/TiO_2_ photocatalysts. The powder samples were dispersed in ethanol and dropped onto Cu grids for analysis. The morphology of photocatalyst was investigated by field emission scanning electron microscopy (FE-SEM, SU8010) that is equipped with an energy dispersive spectrometer (EDS). The surface chemical compositions and oxidation state of elements of the Pd/TiO_2_ and LD-Pd/TiO_2_ photocatalysts were analyzed by X-ray photoelectron spectroscopy (XPS) using an Al Kα X-ray source (Thermo Fisher Scientific). Survey scans were measured from 1200 to -10 eV using a pass energy of 160 eV with a step size of 1 eV and a dwell-time of 0.1 s, whereas the region-of-interest spectra (C1s, O1s, Ti2p, Pd3d and Si2p) were collected using a pass energy of 40 eV with a step size of 0.1 eV and a dwell time of 0.5 s. The adventitious carbon was used for calibration (C1s = 284.6 eV). photoluminescence spectra (PL) were performed using an Edinburgh FLS1000 spectrometer, using a 323 nm laser as the excitation source. X-ray diffraction (XRD) patterns were recorded on a Bruker D8 Advance diffractometer in the scan range of 10 − 70° with a step size of 0.02^o^ and an integration time of 0.2 s using a Cu- Kα radiation source (40 kV, 40 mA). Diffuse reflectance spectra (DRS) of the photocatalysts were measured using an UV-vis spectrophotometer (UV 2600, Shimadzu) equipped with an integrating sphere in the range of 200-800 nm employing BaSO_4_ as the reference. The results are shown in Figs. S5-S8 and Table S1.

**Note S4. Photocatalytic performance**

A mixture of 5 μL KOH (4 M), 34 μL dioxane, 0.5 mL DI water, and 1.5 mL nonane was added into a 4 mL glass reactor. Subsequently, 0.2 mmol quintozene (C_6_Cl_5_NO_2_) and 15 mg photocatalyst were added into the liquid under continuous stirring. The emulsion was purged for 1.5 min by nitrogen gas to remove oxygen. A 365 nm annular LED was used as the light source (38 mW·cm^-2^). A heat sink and a fan were equipped on the LED source to minimize the heat input to the reactor. The 4 mL glass vail was sealed and immersed in a water bath to keep a constant temperature at 20 ^o^C throughout the reaction. The emulsion was demulsified by centrifugation at desired irradiation times, and the aqueous and oil phases were extracted to analyze the quantity of H_2_O_2_ and pentachloroaniline, respectively. In addition to the colorimetric titration method, the concentration of H_2_O_2_ has been also determined by a standard titration method using potassium permanganate to validate the accuracy of the colorimetric titration method (Table S2).^[3]^

Photocatalytic H_2_O_2_ evolution at scaled volumes (20, 60, and 100 mL) were perform using a similar protocol. The volume ratio of water/nonane was fixed at 1:3, and the loading of photocatalyst was kept at 7.5 g·L^−1^. The emulsion was purged for 10 min by nitrogen gas under continuous stirring. Three 365 nm annular LED sources were stacked to realize a homogeneous irradiation of the emulsion.

A comparison with the state-of-the-art results is shown in Table S3.

A real-time video directly compares the LD-Pd/TiO_2_ and Pd/TiO_2_ in decomposition of H_2_O_2_ (Movie S2).

The effect of coating thickness on the photocatalytic performance is evaluated by modifying the Pd/TiO_2_ with variable quantity of LA and DTMS, as shown in Fig. S9.

Note S5. Estimation of quantum efficiency (QE)

The QE for photocatalytic generation of H_2_O_2_ is estimated according to Eq. S1:

$QE=\frac{n_{product}\times n_{e,i}\times N_{A}}{N_{1h}\times t_{R}}\times100 \%$ (1)

where n_product_ is the amount of produced H_2_O_2_ (mol) determined from titration, n_e,i_ is the number of transferred electrons to generate one H_2_O_2_ molecule from water oxidation (2). N_A_ is the Avogadro's constant (6.02×10^23^ mol^−1^), t_R_ is the irradiation time.

The number of incident photons per hour (N_1h_) is calculated according to Eq. S2:

$N_{1h}=\frac{E\times\lambda\times t}{h\times c}=\frac{W_{lamp}\times S_{R}\times\lambda\times t}{(6.626\times{10}^{-34} J\cdot s)\times(3\times{10}^{8} m\cdot s^{1})}$ (2)

where W_lamp_ is the light intensity of LED (38 mW·cm^−2^), S_R_ is the effective irradiation area (1.8 cm^2^ for 2 mL reaction volume). For the reaction operated at 100 mL, the S_R_ is large enough to absorb all incident light, thus the maximum output power of the light (365 mW) was used. *λ* is the wavelength of the light (365 nm), t is irradiation time (3600 s), *h* is the Planck constant (6.626 × 10^-34^ J·s) and *c* is the speed of light (3.0 × 10^8^ m·s^−1^). The estimated N_1h_ of the 2 mL system and 100 mL systems are 4.5×10^20^ and 2.4×10^21^, respectively.

Note S6. Probing the evolution of photogenerated active species

The evolution of photogenerated H atoms and ^•^OH radicals in aqueous phase was quantitively analyzed using 2,2,6,6-tetramethylpiperidinyloxy (TEMPO) as the trap. TEMPO reacts with H atoms and ^•^OH radicals, resulting in the formation of TEMPO-H and TEMPO-OH,^[4]^ as described in Eqs. S3 and S4:


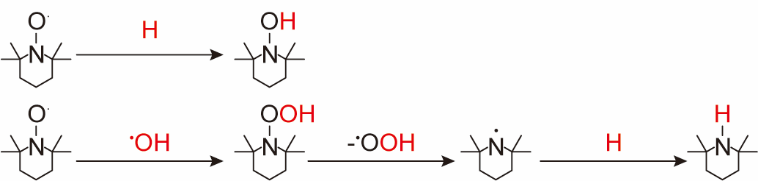
 (3)


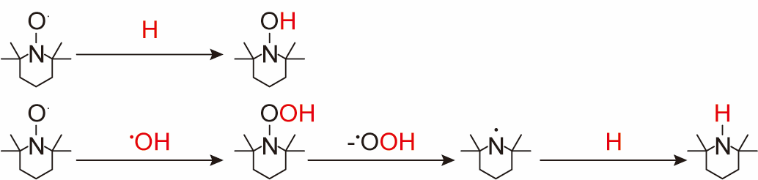
 (4)

The photogenerated TEMPO-H and TEMPO-OH are confirmed by GC-MS, and quantification is realized by GC using standard TEMPO-H and TEMPO-OH samples (Fig. S10).

Practically, a mixture that contains nonane (1.5 mL), water (0.5 mL), KOH (5 μL, 4 M), dioxane (34 μL), and photocatalyst (15 mg) was added into a 4 mL glass reactor, and stirred continuously under N_2_ atmosphere. Subsequently, TEMPO (0.2 mmol) was introduced into the mixture prior to irradiation. The emulsion was then subjected to irradiation, and was demulsified at designated time intervals to collect the aliquots for analysis.

Supplementary Movies


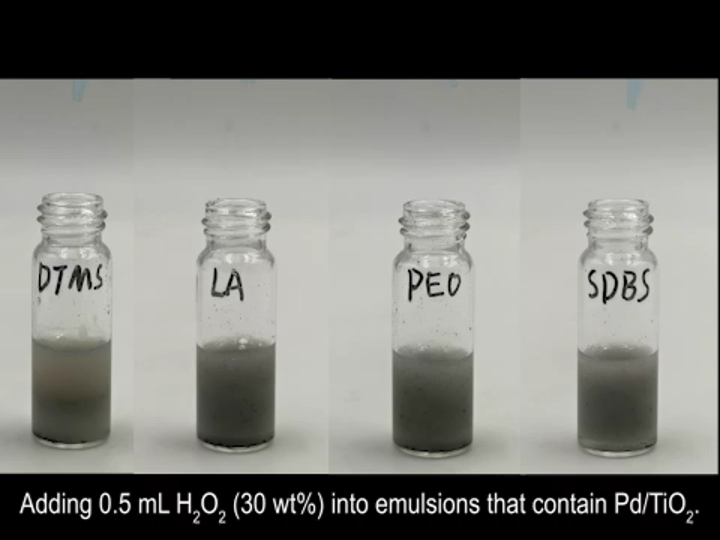


**Movie S1.** Effect of different emulsifiers on Inhibiting catalytic dissociation of H_2_O_2_. A 0.5 mL of concentrated H_2_O_2_ (30 wt%) is added into a 4 mL glass vial that contains 10 mg emulsifier, 0.5 mL water, 1.5 mL nonane, 15 mg Pd/TiO_2_, and 40 mM KOH at RT.


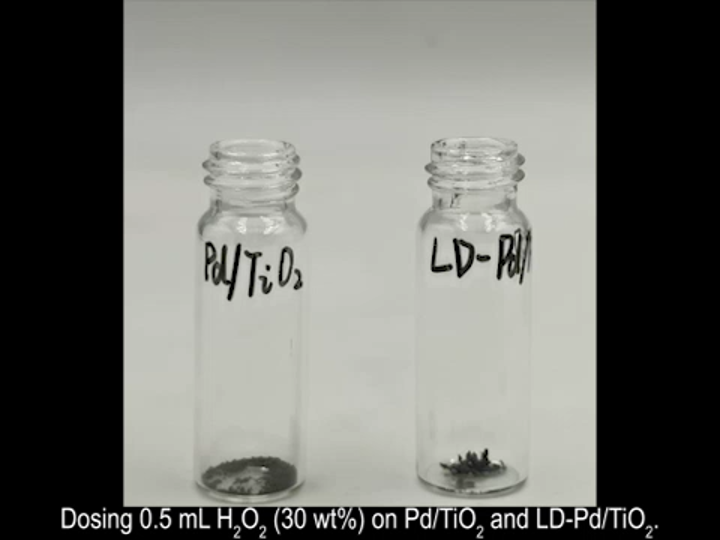


**Movie S2.** Effect of LD-Pd/TiO_2_ on Inhibiting catalytic dissociation of H_2_O_2_ in comparison with uncoated Pd/TiO_2_. A 0.5 mL of concentrated H_2_O_2_ (30 wt%) is added into a 4 mL glass vial that contains 15 mg catalyst.

Supplementary Tables

**Table S1.** Inductively coupled plasma atomic emission spectroscopy (ICP-AES) analysis of metal loadings of Pd/TiO_2_, fresh LD-Pd/TiO_2_, LD-Pd/TiO_2_ after the first cycle (C1), after the six cycles (C6) and after 50 h.

| Catalyst | Metal / wt% | Preparation method for ICP samples |
| --- | --- | --- |
| Pd/TiO_2_-fresh | 2.0 | Direct dissolution in HNO_3_ and HF |
| LD-Pd/TiO_2_-fresh | 1.3 | Sonication in acetone for 3 h, dried in oven, dissolved in HNO_3_ and HF |
| LD-Pd/TiO_2_-C1 | 1.0 |  |
| LD-Pd/TiO_2_-C6 | 0.8 |  |
| LD-Pd/TiO_2_-50 h | 1.2 | Soak in acetone for 6 h, dried in oven, dissolved in HNO_3_ and HF |

**Table S2.** Concentrations of H_2_O_2_ determined by colorimetric analysis and titration.

| Sample ID | No. | [H_2_O_2_] / mM  by colorimetry | [H_2_O_2_] / mM  by titration |
| --- | --- | --- | --- |
| 100 mM  H_2_O_2_ solution | Test 1 | 98.3 | 107.0 |
|  | Test 2 | 99.9 | 109.0 |
| Reaction  solution | Test 1 | 132.9 | 140.0 |
|  | Test 2 | 133.2 | 142.0 |

**Table S3.** Comparison of reported H_2_O_2_ production *via* WOR.^[5]^


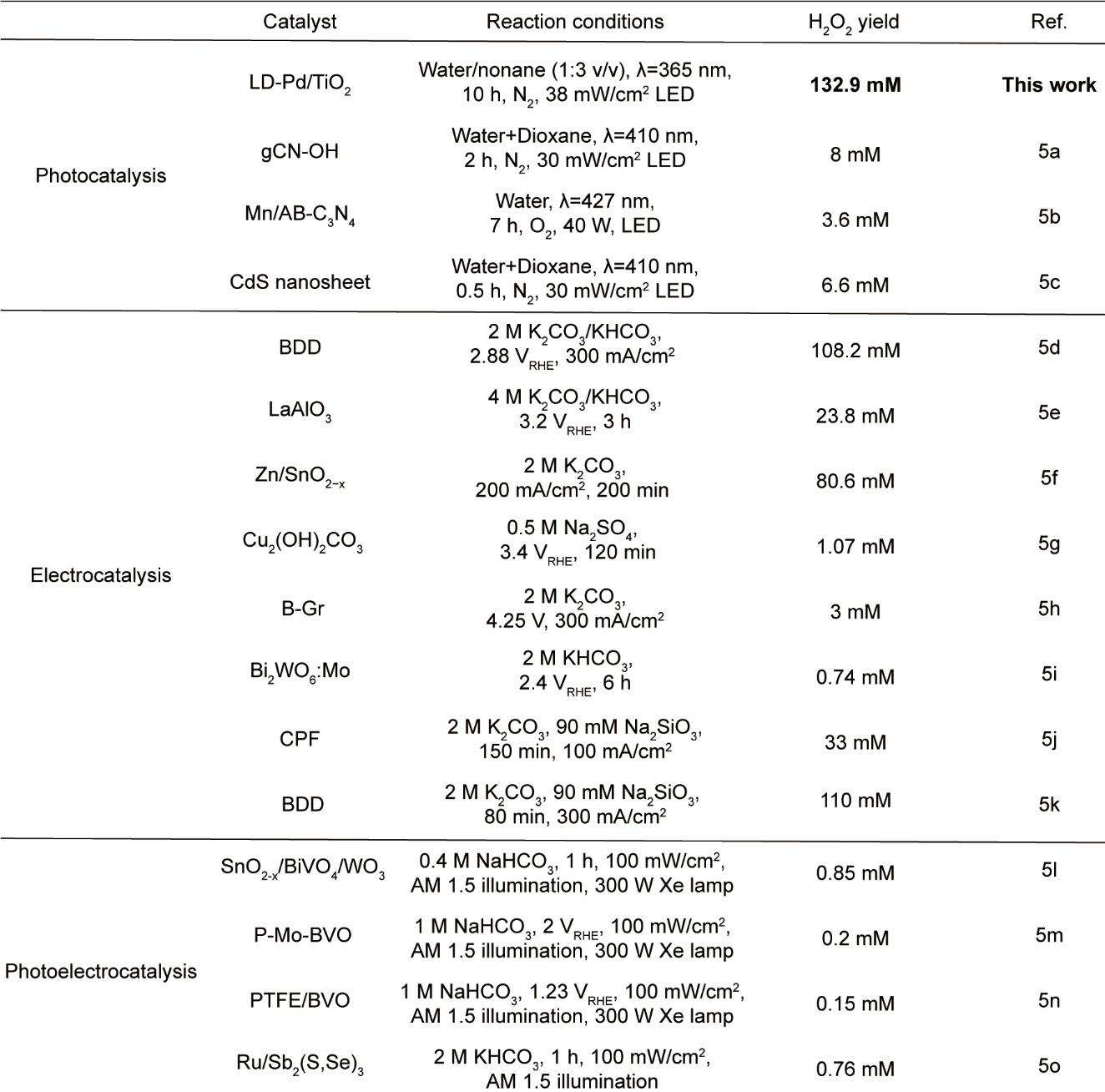


Supplementary Figures


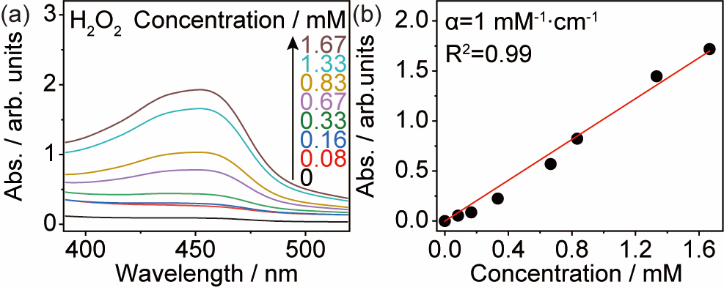


**Figure S1.** Colorimetric titration of H_2_O_2_. (a) UV-vis spectra and (b) derived absorption coefficient of Cu(I)-DMP complex at 454 nm.


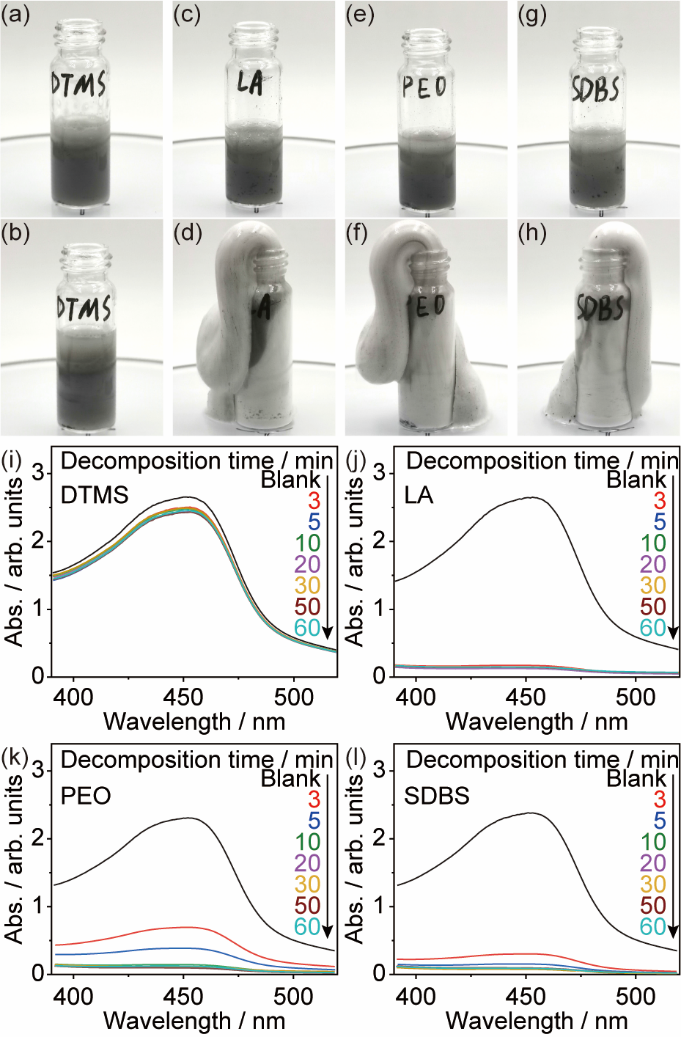


**Figure S2.** Effect of emulsifier in supressing H_2_O_2_ decomposition. (a)-(d) Images of water-nonane-Pd/TiO_2_ with DTMS, LA, PEO, and SDBS before and after dosing H_2_O_2_ under ambient conditions; (e)-(h) UV-vis spectra for the colorimetric titration of H_2_O_2_ in water with the presence of DTMS, LA, PEO, and SDBS emulsifiers.


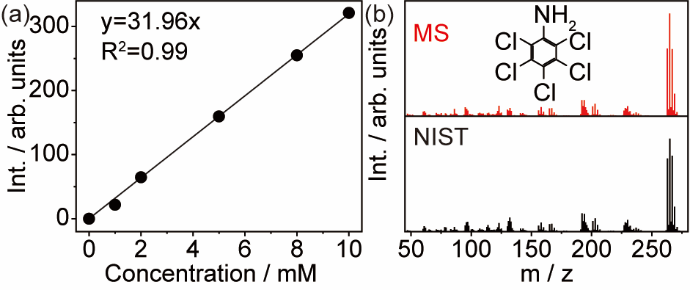


**Figure S3.** Identification and quantification of hydrogenated quintozene. (a) calibration curves and (b) MS spectra of pentachloroaniline (C_6_Cl_5_NH_2_) by GC.


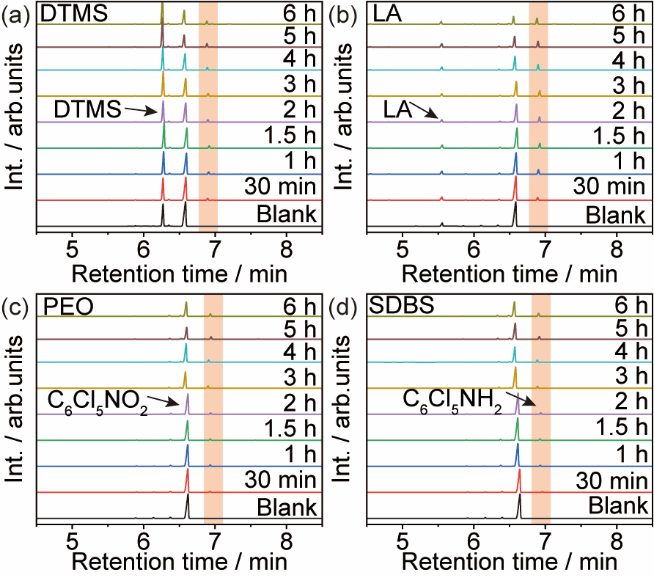


**Figure S4.** Effect of emulsifier in promoting photocatalytic hydrogenation of quintozene (C_6_Cl_5_NO_2_). (a)-(d) GC spectra of separated oil phase using DTMS, LA, PEO, and SDBS as emulsifiers.


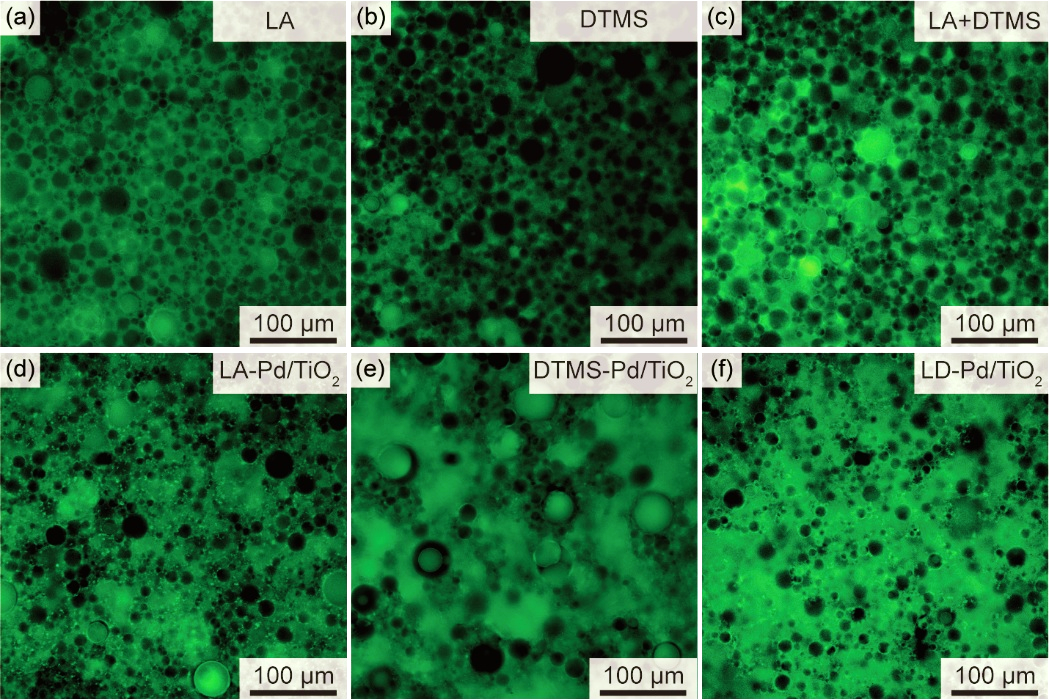


**Figure S5.** Microscopic imaging of the W/O emulsion with (a) LA, (b) DTMS, (c) LA+DTMS, (d) LA-Pd/TiO_2_, (e) DTMS-Pd/TiO_2_, and (f) LD-Pd/TiO_2_.


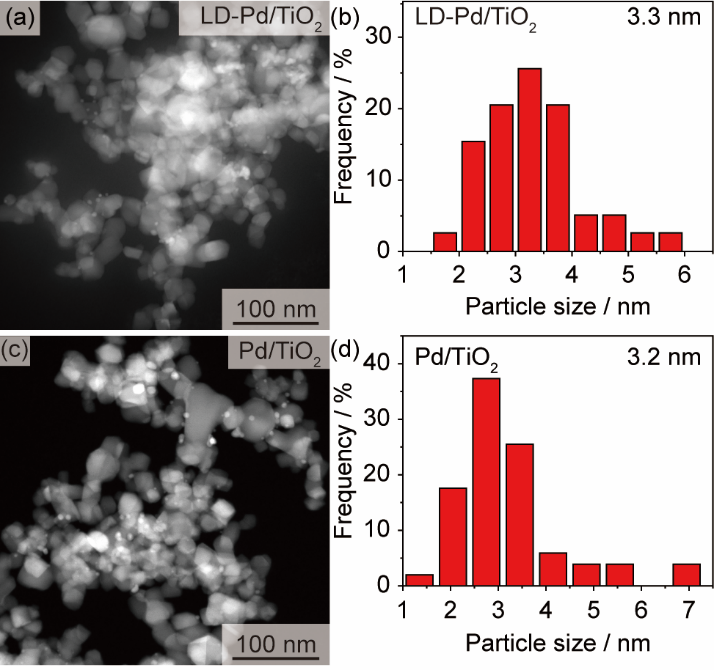


**Figure S6.** (a) TEM imaging and (b) particle size distribution of Pd nanoparticles in LD-Pd/TiO_2_, (c) TEM imaging and (d) particle size distribution of the Pd/TiO_2_.

**
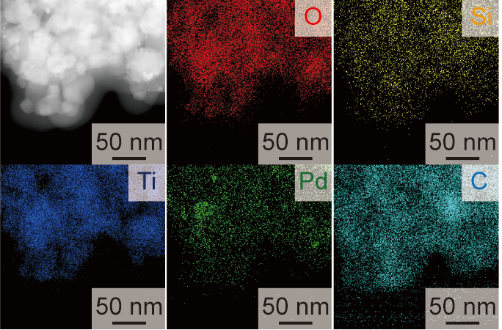
**

**Figure S7.** EDS mapping of the LD-Pd/TiO_2_.


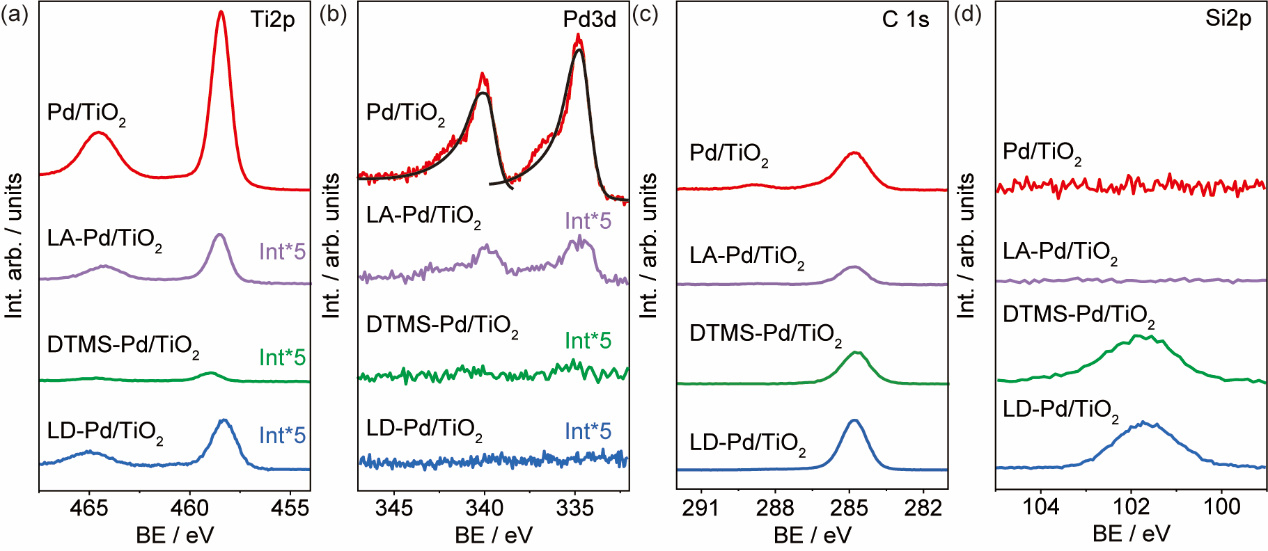


**Figure S8.** (a)-(d) XPS of the Pd/TiO_2_, LA-Pd/TiO_2_, DTMS-Pd/TiO_2_ and LD-Pd/TiO_2_.


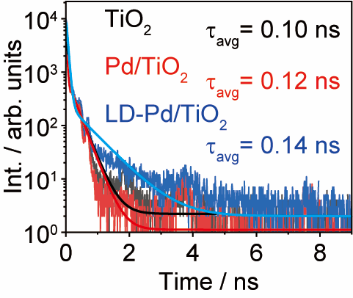


**Figure S9.** Normalized transient PL decay profiles for TiO_2_, Pd/TiO_2_ and LD-Pd/TiO_2_.

The diffuse reflectance spectroscopy (DRS) results reveal that the optical bandgap of the LD-Pd/TiO_2_, LA-Pd/TiO_2_, DTMS-Pd/TiO_2_, Pd/TiO_2_ and TiO_2_ are similar (~3.2 eV, Fig. S10). Mott-Schottky analysis indicates that the flat-band potentials (V_Fb_) for the LD-Pd/TiO_2_ is -0.7 V *vs.* RHE, the conduction band minimum (CBM) and valence band maximum (VBM) of the LD-Pd/TiO_2_ all experience a negative shift (-0.3 V) compared to Pd/TiO_2_ and TiO_2_. In addition, the CBM and VBM of LA-Pd/TiO_2_ and DTMS-Pd/TiO_2_ are very close to LD-Pd/TiO_2_.


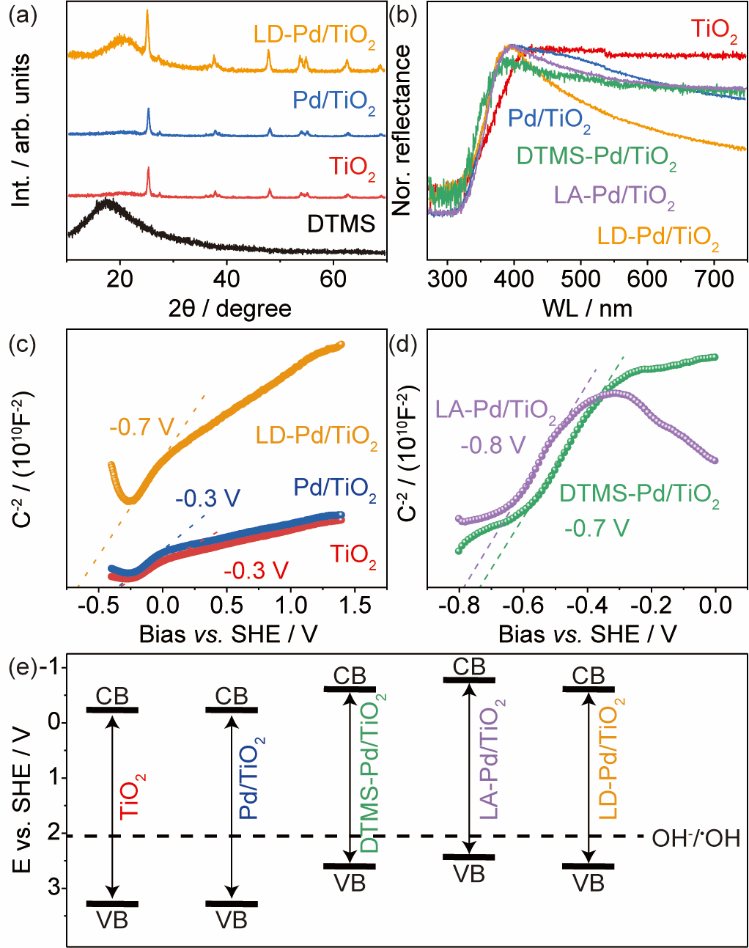


**Figure S10.** (a) XRD, (b) DRS, (c) and (d) Mott-Schottky plots and (e) Derived band positions of DTMS, TiO_2_, Pd/TiO_2_, LA-Pd/TiO_2_, DTMS-Pd/TiO_2_ and LD-Pd/TiO_2_.


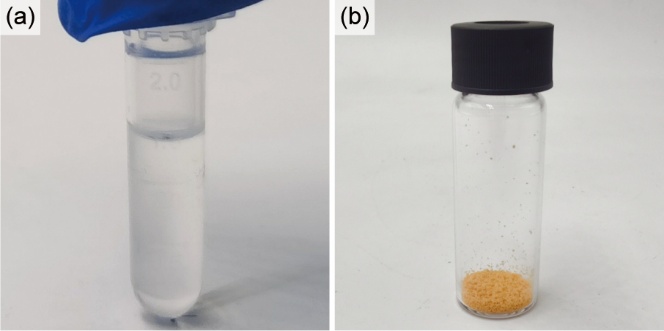


**Figure S11.** Images of (a) recovered nonane and (b) pentachloroaniline by rotary evaporation of the separated oil phase.


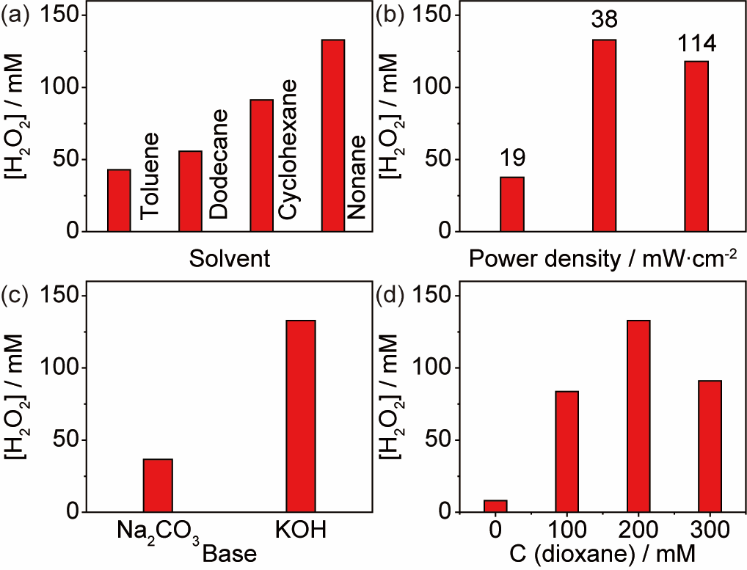


**Figure S12.** Effect of (a) solvent, (b) light intensity, (c) base, and (d) concentration of dioxane on photocatalytic H_2_O_2_ evolution. Reaction conditions:15 mg LD-Pd/TiO_2_ in 0.5 mL water and 1.5 mL solvent with 100 mM quintozene, 1,4-dioxane and 40 mM base under 365 nm irradiation and 1 bar N_2_ at RT for 10 h.


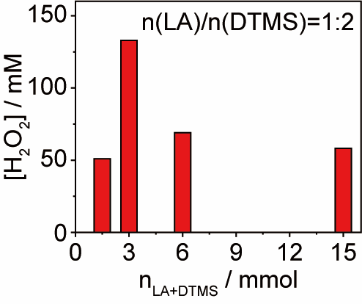


**Figure S13.** Photocatalytic H_2_O_2_ production from water dissociation using LD-Pd/TiO_2_ with variable quantity of LA and DTMS coated on Pd/TiO_2_. Reaction conditions: 15 mg photocatalyst in 0.5 mL water and 1.5 mL nonane with 100 mM quintozene, 1.7 vol% 1,4-dioxane and 40 mM KOH under 365 nm irradiation (38 mW∙cm^‒2^) and 1 bar N_2_ at RT for 10 h.


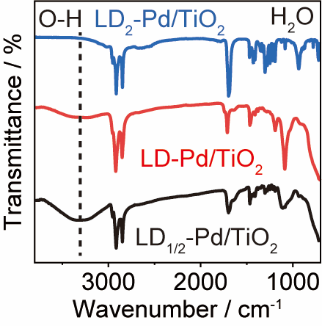


**Figure S14.** FTIR of the LD_1/2_-Pd/TiO_2_, LD-Pd/TiO_2_ and LD_2_-Pd/TiO_2_ with H_2_O. The subscripts “1/2” and “2” denote the relative loadings of LA and DTMS to the standard quantity.


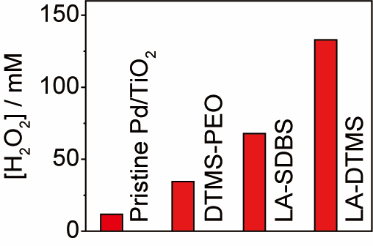


**Figure S15.** Photocatalytic H_2_O_2_ production from water oxidation using DTMS-PEO and LA-SDBS coated Pd/TiO_2_, in comparison with pristine Pd/TiO_2_ and LA-DTMS coated Pd/TiO_2_ (LD-Pd/TiO_2_). Reaction conditions: 15 mg photocatalyst in 0.5 mL water and 1.5 mL nonane with 100 mM quintozene, 1.7 vol% 1,4-dioxane and 40 mM KOH under 365 nm irradiation (38 mW∙cm^‒2^) and 1 bar N_2_ at RT for 10 h.


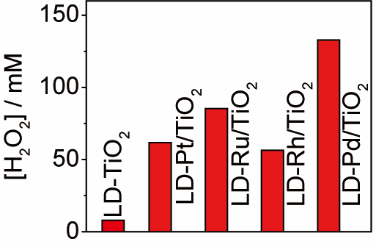


**Figure S16.** Photocatalytic H_2_O_2_ evolution from water dissociation using emulsifier coated TiO_2_ with different metal cocatalysts. Standard reaction conditions: 15 mg photocatalyst in 0.5 mL water and 1.5 mL nonane with 100 mM quintozene, 1.7 vol% 1,4-dioxane and 40 mM KOH under 365 nm irradiation (38 mW∙cm^‒2^) and 1 bar N_2_ at RT for 10 h.


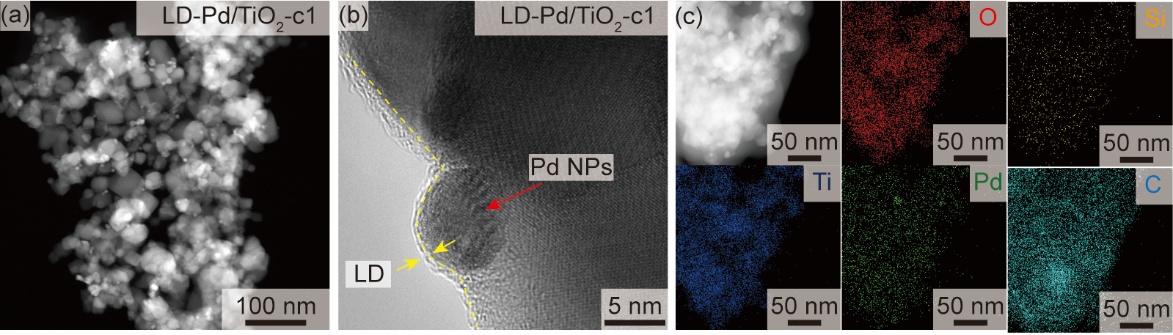


**Figure S17.** (a) and (b) TEM imaging and (c) EDS mapping of the LD-Pd/TiO_2_-C1.


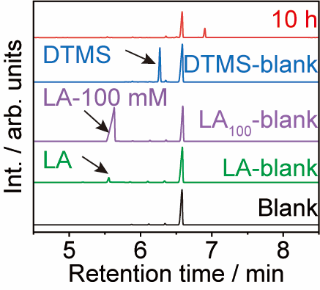


**Figure S18.** GC spectra of the separated oil phase containing quintozene, LA, and DTMS in comparison with an irradiated emulsion with LD-Pd/TiO_2_ (10 h).


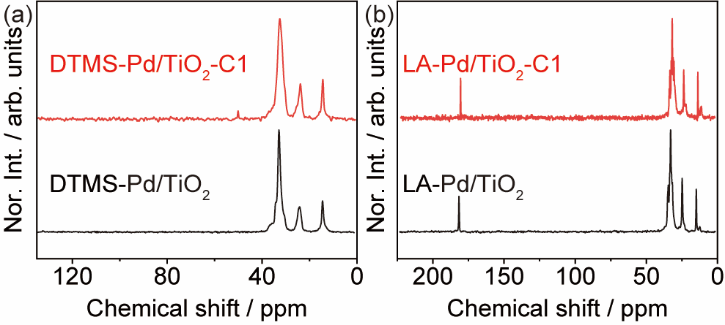


**Figure S19.** ^13^C ssNMR spectra of (a) DTMS-Pd/TiO_2_ and DTMS-Pd/TiO_2_-C1, (b) LA-Pd/TiO_2_ and LA-Pd/TiO_2_-C1.

No H_2_O_2_ was generated when 5 M H_2_SO_4_ (5 µL) was added or under neutral conditions (Fig. S20), suggesting that the generation of H_2_O_2_ occurs *via* an indirect water oxidation pathway, where the ^•^OH radicals are produced by the oxidation of OH^‒^ anions in an alkaline medium.


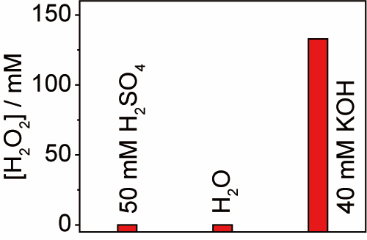


**Figure S20.** The catalytic performance of LD-Pd/TiO_2_ in acidic, neutral, and basic medium. Reaction conditions: 15 mg photocatalyst in 0.5 mL water and 1.5 mL nonane with 100 mM quintozene, 1.7 vol% 1,4-dioxane, and 5 µL solution (5 M H_2_SO_4_, H_2_O, or 4 M KOH) under 365 nm irradiation (38 mW·cm^−2^) and 1 bar N_2_ at RT for 0.5 h.


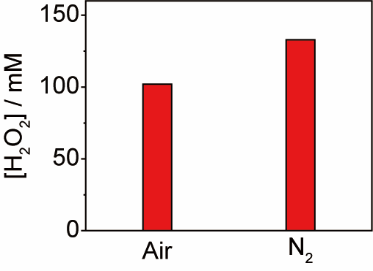


**Figure S21.** Photocatalytic H_2_O_2_ production from water oxidation under air and N_2_ atmospheres.


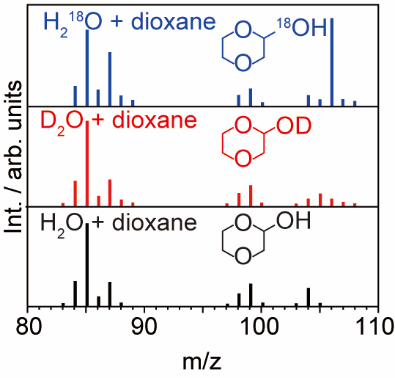


**Figure S22.** MS analysis of generated 1,4-dioxane-2-ol during irradiation of LD-Pd/TiO_2_ in acidic media using H_2_O, D_2_O, and H_2_^18^O. Reaction conditions: 15 mg photocatalyst in 2 ml nonane/water (3:1 v/v), 1.7 vol% dioxane and 5 µL 5 M H_2_SO_4_ with 100 mM C_6_Cl_5_NO_2_ under 365 nm irradiation (38 mW∙cm^-2^) and 1 bar N_2_ at RT for 10 h.


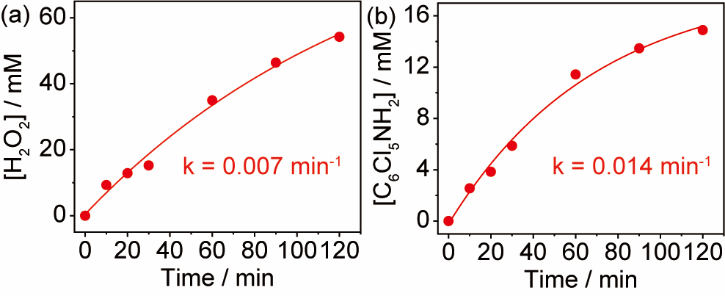


**Figure S23.** Kinetics of photocatalytic (a) H_2_O_2_ evolution and (b) pentachloroaniline production from quintozene reduction in emulsion with dioxane (0.25 mL) and water (0.25 mL). Reaction conditions: 15 mg photocatalyst, 1.5 mL nonane with 100 mM quintozene and 40 mM KOH under 365 nm irradiation (38 mW∙cm^‒2^) under 1 bar N_2_ at RT.


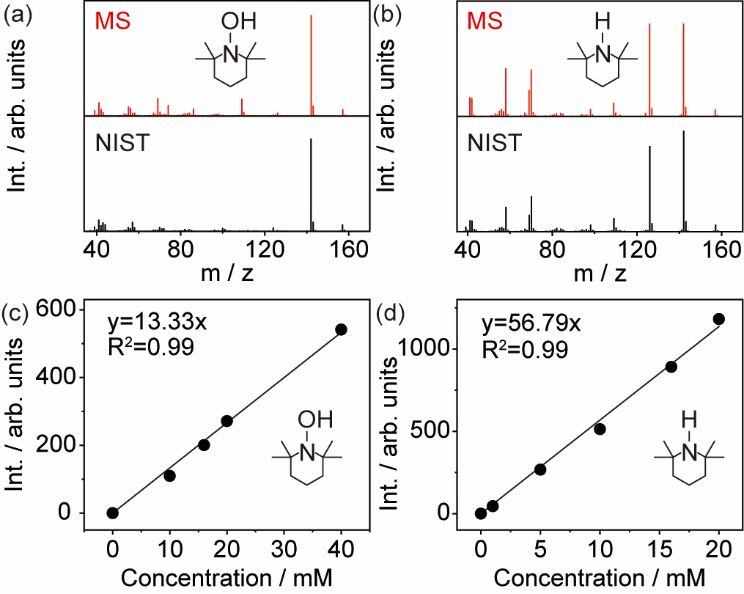


**Figure S24.** (a)-(d) GC-MS characterizations and calibration curves of TEMPO-H and TEMPO-OH.


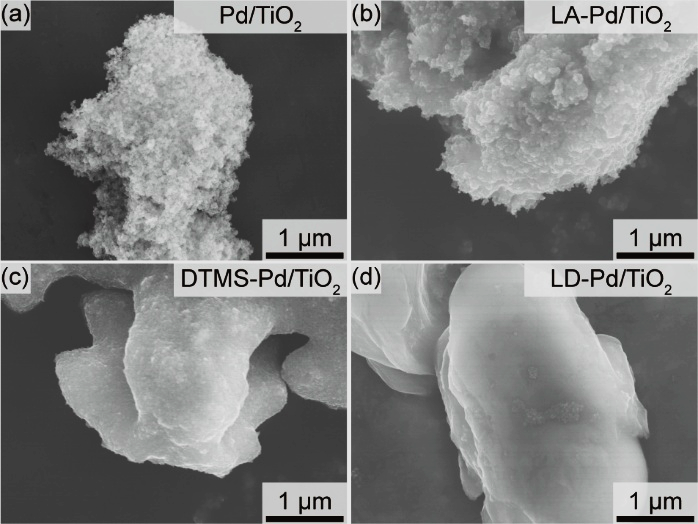


**Figure S25.** (a)-(d) SEM imaging of the Pd/TiO_2_, LA-Pd/TiO_2_, DTMS-Pd/TiO_2_ and LD-Pd/TiO_2_.


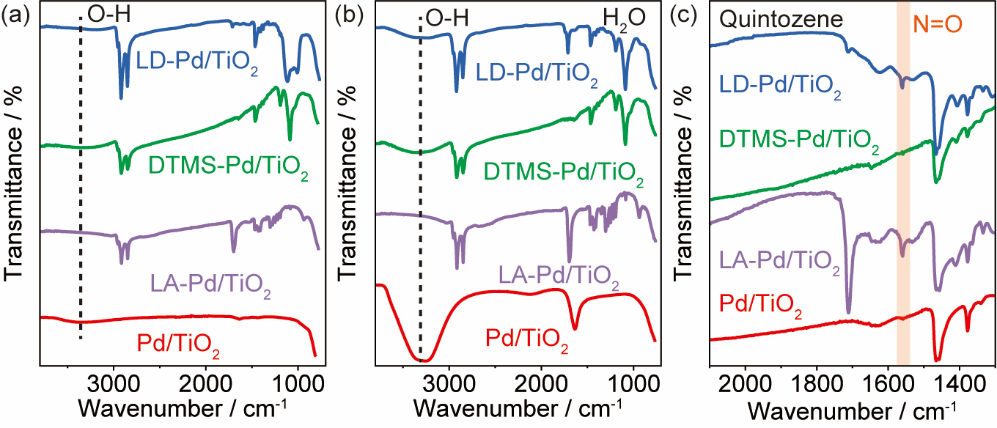


**Figure S26.** FTIR of Pd/TiO_2_, LA-Pd/TiO_2_, DTMS-Pd/TiO_2_, and LD-Pd/TiO_2_. (a) dried, (b) and (c) with the addition of H_2_O and quintozene.


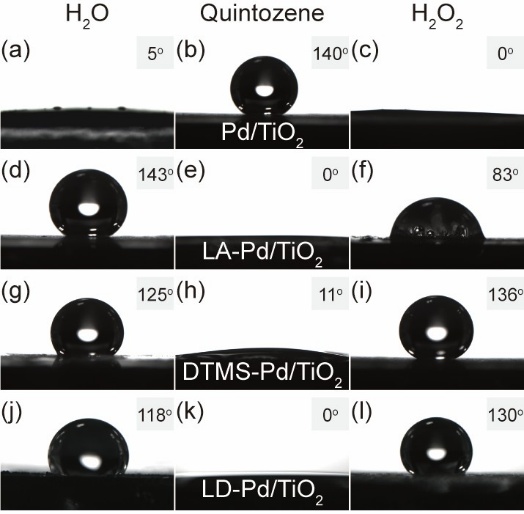


**Figure S27.** Contact angles of water, quintozene, and H_2_O_2_ on Pd/TiO_2_ (a)-(c), LA-Pd/TiO2 (d)-(f), DTMS-Pd/TiO_2_ (g)-(i), and LD-Pd/TiO_2_ (j)-(l).

# References

[1] A. N. Baga, G. R. A. Johnson, N. B. Nazhat, R. A. Saadalla-Nazhat, *Anal. Chim. Acta* **1988**, *204*, 349-353.

[2] W. Qiao, X. Fan, W. Liu, F. N. Khan, D. Zhang, F. Han, H. Yue, Y. Li, N. Dimitratos, S. Albonetti, X. Wen, Y. Yang, F. Besenbacher, Y. Li, H. Niemantsverdriet, H. Lin, R. Su, *J. Am. Chem. Soc.* **2023**, *145*, 5353-5362.

[3] T. He, H. Tang, J. Wu, J. Wang, M. Zhang, C. Lu, H. Huang, J. Zhong, T. Cheng, Y. Liu, Z. Kang, *Nat. Commun.* **2024**, *15*, 7833.

[4] Q. Wu, J. Ye, W. Qiao, Y. Li, J. W. Niemantsverdriet, E. Richards, F. Pan, R. Su, *Appl. Catal., B* **2021**, *291*, 120118.

[5] a) D. Zhang, P. Ren, W. Liu, Y. Li, S. Salli, F. Han, W. Qiao, Y. Liu, Y. Fan, Y. Cui, Y. Shen, E. Richards, X. Wen, M. H. Rummeli, Y. Li, F. Besenbacher, H. Niemantsverdriet, T. Lim, R. Su, *Angew. Chem. Int. Ed.* **2022**, *61*, e202204256; b) P. Ren, T. Zhang, N. Jain, H. Y. V. Ching, A. Jaworski, G. Barcaro, S. Monti, J. Silvestre-Albero, V. Celorrio, L. Chouhan, A. Rokicińska, E. Debroye, P. Kuśtrowski, S. Van Doorslaer, S. Van Aert, S. Bals, S. Das, *J. Am. Chem. Soc.* **2023**, *145*, 16584-16596; c) Y. Mai, D. Zhang, K. Maliutina, X. Leng, N. Cai, J. Li, C. Wang, Y. Huang, K. Zhang, W. Zhang, Y. Li, F. Besenbacher, H. Niemantsverdriet, W. Liang, Y. Shen, T. Lim, E. Richards, R. Su, *Adv. Sci.* **2025**, *12*, 2410680; d) S. Mavrikis, M. Göltz, S. C. Perry, F. Bogdan, P. K. Leung, S. Rosiwal, L. Wang, C. Ponce de León, *ACS Energy Lett.* **2021**, *6*, 2369-2377; e) J. Baek, Q. Jin, N. S. Johnson, Y. Jiang, R. Ning, A. Mehta, S. Siahrostami, X. Zheng, *Nat. Commun.* **2022**, *13*, 7256; f) X. Hu, H. Jiang, R. Chen, L.-M. Yang, X. Li, B. Y. Xia, B. You, *ACS Catal.* **2025**, *15*, 8403-8413; g) R. Wang, H. Luo, C. Duan, H. Liu, M. Sun, Q. Zhou, Z. Ou, Y. Lu, G. Luo, J. C. Yu, Z. Hu, *Nat. Commun.* **2024**, *15*, 10456; h) U. Javed, M. Tebyetekerwa, C. Tang, X. Zeng, Z. Wang, K. Sun, J. Yang, I. Marriam, L. Guo, X. Sun, A. K. Sahu, Y. Zhang, A. Zamyadi, A. Du, Q. Li, T. E. Rufford, X. Zhang, *Adv. Mater.* **2025**, *2500834*, 2500834; i) L. Li, Z. Hu, J. C. Yu, *Angew. Chem. Int. Ed.* **2020**, *59*, 20538-20544; j) D. Pangotra, L.-I. Csepei, A. Roth, C. Ponce de León, V. Sieber, L. Vieira, *Appl. Catal., B* **2022**, *303*, 120848; k) D. Pangotra, L.-I. Csepei, A. Roth, V. Sieber, L. Vieira, *Green Chem.* **2022**, *24*, 7931-7940; l) C. Dong, Y. Yang, X. Hu, Y. Cho, G. Jang, Y. Ao, L. Wang, J. Shen, J. H. Park, K. Zhang, *Nat. Commun.* **2022**, *13*, 4982; m) T. H. Jeon, H. Kim, H.-i. Kim, W. Choi, *Energy Environ. Sci.* **2020**, *13*, 1730-1742; n) M. Ou, M. Geng, X. Fang, W. Shao, F. Bai, S. Wan, C. Ye, Y. Wu, Y. Chen, *Adv. Sci.* **2023**, *10*, 2300169; o) L. Wang, F. Guo, S. Ren, R.-T. Gao, L. Wu, *Angew. Chem. Int. Ed.* **2024**, *63*, e202411305.
